# Cav3.2 calcium channel interactions with the epithelial sodium channel ENaC

**DOI:** 10.1186/s13041-019-0433-8

**Published:** 2019-02-08

**Authors:** Agustin Garcia-Caballero, Maria A. Gandini, Shuo Huang, Lina Chen, Ivana A. Souza, Yan L. Dang, M. Jackson Stutts, Gerald W. Zamponi

**Affiliations:** 1 Molecular Neuroscience, Department of Physiology and Pharmacology, Hotchkiss Brain Institute and Alberta Children’s Hospital Research Institute, Cumming School of Medicine, University of Calgary, 3330 Hospital Dr. NW, Calgary, T2N 4N1 Canada; 2 Cystic Fibrosis Center, University of North Carolina at Chapel Hill, Chapel Hill, NC USA

**Keywords:** Cav3.2 calcium channels, Epithelial Sodium Channel (ENaC), Dorsal root ganglia (DRG), Dorsal horn

## Abstract

This study describes the functional interaction between Cav3.2 calcium channels and the Epithelial Sodium Channel (ENaC). β-ENaC subunits showed overlapping expression with endogenous Cav3.2 calcium channels in the thalamus and hypothalamus as detected by immunostaining. Moreover, β- and γ-ENaC subunits could be co-immunoprecipitated with Cav3.2 calcium channels from brain lysates, dorsal horn and lumbar dorsal root ganglia. Mutation of a cluster of lysines present in the intracellular N-terminus region of β-ENaC (K4R/ K5R/ K9R/ K16R/ K23R) reduced interactions with Cav3.2 calcium channels. Αβγ-ENaC channels enhanced Cav3.2 calcium channel trafficking to the plasma membrane in tsA-201 cells. This effect was reciprocal such that Cav3.2 channel expression also enhanced β-ENaC trafficking to the cell surface. T-type current density was increased when fully assembled αβγ-ENaC channels were transiently expressed in CAD cells, a neuronal derived cell line. Altogether, these findings reveal ENaC as an interactor and potential regulator of Cav3.2 calcium channels expressed in neuronal tissues.

**Electronic supplementary material:**

The online version of this article (10.1186/s13041-019-0433-8) contains supplementary material, which is available to authorized users.

## Introduction

The Epithelial Sodium Channel (ENaC) is a key regulator of sodium absorption in a variety of tissues. It fine tunes sodium in the nephron and the colonic epithelia [1] where it helps to maintain total body salt and volume homeostasis. In the pulmonary airway epithelia ENaC regulates the composition and depth of the airway surface liquid to maintain mucociliary clearance [2, 3]. Malfunction of ENaC in these tissues results in various cardiovascular [4] and lung diseases, such as cystic fibrosis [5]. ENaC typically consists of three subunits (α, β, and γ) which when co-assembled form a sodium selective pore [6]. Each subunit is composed of two transmembrane domains with an amino-terminal (N-terminal) cytosolic domain, a large extracellular loop and a cytosolic carboxyl-terminus (C-terminus) [3]. Gain or loss of function mutations in all ENaC subunits result in disorders such as hypertension or Liddle’s Syndrome and pseudohypoaldosteronism [4, 7]. Importantly, ENaC is inhibited by the Cystic Fibrosis transmembrane conductance regulator (CFTR) via channel / channel interactions [8]. ENaC can also interact with the thiazide-sensitive sodium chloride cotransporter (NCC) in the kidney [9], suggesting that these channels can readily interact with other membrane proteins. ENaC expression is not limited to non-neuronal tissues as evident from reported expression in mechanosensory neurons [10], dorsal root ganglia (DRG) [11] and brain. However, the function of ENaC channels in neurons has not been well described.

Certain types of sensory neurons express Cav3.2 calcium channels [12, 13] These channels belong to the family of low-voltage gated T-type calcium channels [14]. Their biophysical properties, such as low voltage thresholds for activation and inactivation, fast inactivation and rebound bursting, are important for neuronal excitability in both the central and peripheral nervous systems [14, 15]. Cav3.2 calcium channels are also expressed in the thalamus [15] where they play an important role in epilepsy [16–18]. Importantly, Cav3.2 calcium channels can form channel complexes with members of the potassium channel family such as Kv4, KCa1.1, and KCa3.1 to regulate neuronal activity [19–21].

Because of the overlap in ENaC and Cav3.2 channel expression in sensory neurons, and the reported inhibitory effect of intracellular calcium on the open probability of ENaC and on ENaC expression [22–24], we tested whether Cav3.2 channels may form a protein complex with ENaC. We find that specific ENaC channel subunits interact with Cav3.2 channels and reciprocally regulate each other’s membrane expression.

## Methods

### Cell culture and transfection

Human embryonic kidney tsA-201 cells were cultured as described previously [25]. Cells were transfected with calcium phosphate and used for biochemical and electrophysiological analysis 48–72 h post-transfection. Mouse CAD cells were cultured as described previously [13]. Cells were transfected with lipofectamine 2000 and used for biochemical assays 48–72 h post-transfection.

### Plasmids

cDNAs encoding rat α-, β-, and γ-ENaC with HA–N-terminal (HA-NT) epitope tags were used. WT and mutant constructs (β-ENaC: K4R/ K5R/ K9R/ K16R/ K23R) were generated by PCR. Mutations were performed with the Quik change mutagenesis kit (Stratagene). The WT ENaC plasmids were provided by B. Rossier (Université de Lausanne, Lausanne, Switzerland). The sequence of all plasmids was verified at the University of North Carolina sequencing facility.

### Biochemistry

Co-immunoprecipitation (co-IP) experiments were followed by western blots. All tissue was derived from wildtype C57BL/6 mice. All procedures for tissue isolation were approved by the Animal Care Committee of the University of Calgary. Both lumbar DRG and dorsal horn tissue were lysed in modified RIPA buffer (in mM; 50 Tris, 100 NaCl, 0.2% (*v*/v) Triton X-100, 0.2% (v/v) NP-40, 10 EDTA + protease inhibitor cocktail, pH 7.5). Tissue lysates were used to immunoprecipitate Cav3.2 channels with a specific Cav3.2 polyclonal antibody (Sigma). Lysates were prepared by sonicating samples at 60% pulse for 10 s and by centrifugation at 13,000 rpm for 15 min at 4 °C. Supernatants were transferred to new tubes and solubilized proteins were incubated with 50 ml of Protein G/A beads (Piercenet) and 2 μg of anti-Cav3.2 antibody overnight while tumbling at 4 °C. Cav3.2 immunoprecipitates were washed twice with modified RIPA buffer and beads were aspirated to dryness. Laemmli buffer was added and samples were incubated at 96 °C for 10 min. Eluted samples were loaded on a 7.5% Tris-glycine gel or 10% Tris-glycine gel for inputs and resolved using SDS-PAGE. Samples were transferred to 0.45 mm polyvinylidenedifluoride (PDVF) membranes (Millipore) by dry-transfer with i-blot (Invitrogen). Membranes were probed for α-ENaC (UNC1 19.2.1, 11,000), β-ENaC (Alomone, 1:500) or γ-ENaC subunits (StressMarq Biosciences, 1:500) with specific antibodies. Inputs, representing 4% of total lysate, were probed for alpha-tubulin (Abcam, 1:2000), as loading controls. Densitometry analysis was performed using the ImageJ program. Student T test, *p* < 0.05 was considered significant (*p < 0.05, **p < 0.01 and ***p < 0.001).

### Surface labelling

tsA-201 cells were transfected with either Cav3.2 cDNA alone or in combination with α-, β-, and γ-ENaC subunits cDNA separately, or with all three ENaC subunits together (10 μg per 10-cm dish). Cells grown in poly-lysine plates were washed twice with PBS buffer. Cells were pre-chilled on ice for 20 min and labeled with 0.75 mg ml^− 1^ sulfo-NHS-biotin in PBS-Ca-Mg (mM), 1 MgCl2, 0.1 CaCl2, titrated to pH 8.3 with NaOH, while tumbling gently for 40 min at 4 °C. Cells were washed twice with chilled PBS-Ca-Mg buffer and incubated in PBS-Ca-Mg buffer with 100 mM glycine for 15 min at 4 °C to quench free biotin. Cells were washed again three times with chilled PBS-Ca-Mg buffer, then lysed with lysis buffer (in mM; 50 Tris, 100 NaCl, 1% triton X-100 (vol/vol), 1% NP-40 (vol/vol), 0.2% SDS (wt/vol), 0.1% NaDeoxycholate (wt/vol), 20 NaF, 10 Na_4_P_2_O_7_ pyrophosphate, 10 EDTA + protease inhibitor cocktail (complete, Roche), pH 7.5). Cell lysates were prepared by sonicating samples at 60% pulse intensity for 10 s and by centrifugation at 14,000 rpm for 15 min at 4 °C. Supernatants were transferred to new tubes and solubilized proteins were incubated with 30 μl of neutravidin beads (Pierce) overnight while tumbling at 4 °C. Total inputs were taken from whole cell samples representing 4% of total protein. Samples were washed twice with (mM) 500 NaCl, 50 Tris pH 7.5 buffer and once with 150 NaCl, 50 Tris pH 7.5 buffer. Laemmli buffer was added and samples were loaded on a 10% Tris-glycine gel after incubation for 10 min at 96 °C. Samples were transferred to 0.45 μm polyvinylidene difluoride (PDVF) membranes by dry transfer using an I-blot machine (Invitrogen) and western blot analysis was performed using an anti-Cav3.2 antibody (Sigma) or an anti-β-ENaC antibody (Alomone).

### Immunostaining

Sections were washed in 1X PBS 3 times before blocked for 1.5 h with vehicle (0.5% BSA + 10% NGS + 0.3% Triton X-100 in 1X PBS). Sections were then incubated with primary antibody (Rabbit anti β-ENaC, 1:200, Alomone labs, ASC-019; Mouse anti Cav3.2, 1:200, Novus, NBP1–22444) overnight at 4 °C. Sections were washed 3 times in vehicle before incubation with secondary antibody (Goat anti rabbit, Alexa Fluor 488, 1:800, Invitrogen A11029; Goat anti mouse, Alexa Fluor 633, 1:800, Invitrogen A21070) for 1.5 h at room temperature. Sections were washed 3 times in vehicle, 1X PBS, and 0.5X PBS, respectively before being air dried and mounted on coverslips. Sections were scanned with either a Zeiss LSM 510 or a Leica TCS SP8 X confocal microscope.

### Electrophysiology

Electrophysiological recordings were performed using the whole cell configuration of the patch-clamp technique at room temperature using an Axopatch 200B amplifier (Axon Instruments, Union City, CA). Acquisition was performed using pClamp9 (Axon Instruments) and analysis was performed with Clampfit 9 (Axon Instruments) and GraphPad Prism. For CAD cells the internal recording solution contained (in mM): 110 CsCl, 3 MgATP, 0.5 Na-GTP, 2.5 MgCl_2_, 5 D-glucose, 10 EGTA, 10 HEPES (pH 7.3 with CsOH). The external solution contained (in mM): 10 BaCl_2_, 1 MgCl_2_, 140 TEACl, 10 D-glucose, 10 HEPES (pH 7.2 with TEAOH). Cell with more than 100 pA current leak were not considered. Currents from tsA-201 cells were recorded using an external solution contained (in mM): 5 BaCl_2_, 1 MgCl_2_, 150 TEACl, 10 D-glucose, 10 HEPES (pH 7.2 with TEAOH).

The *I-V* relationships were fitted with a Boltzmann equation of the form: I = Gmax*(Vm − Vr)/(1 + exp(−(Vm − V_*1/2,act*_*)/k)),* where *I* is the peak current density,*Vm* is the membrane voltage, V_*1/2,act*_ is the voltage for half activation, *Vr* is the reversal potential, and *k* is the slope factor. Steady-state inactivation curves were fitted with the equation: *I/Imax = 1/(1 + exp(−(V − V*_*1/2,inac*_*)/k))*, where *I/Imax* is the normalized current, *V* is the conditioning voltage, *V*_*1/2,inac*_ is the voltage for half-inactivation and *k* is the slope factor.

### Statistical analysis

The significance of observed differences was evaluated by Student’s *t* tests and One Way Analysis of Variance as appropriate. A probability less than 5% was considered to be significant.

## Results

### Cav3.2 channels and ENaC subunits interact

**Fig. 1:**
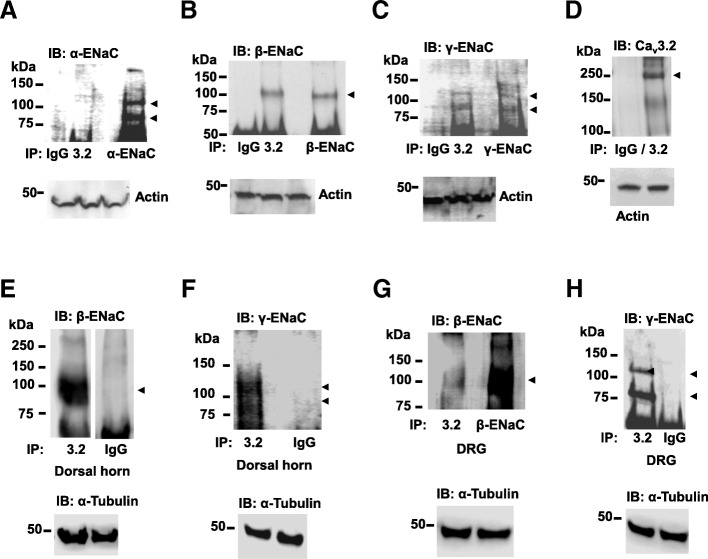
Western blots showing Cav3.2 and ENaC complexes in neuronal tissue. Cav3.2 immunoprecipitates from mouse whole brain lysates were probed for either (**a**) α-, *n* = 3 (**b**) β-, *n* = 3 (**c**) γ-ENaC subunits, *n* = 3 (arrows indicate full-length and cleaved proteins) or (**d**) Cav3.2 calcium channels, *n* = 4. Actin loading controls are shown. (**e**) Cav3.2 immunoprecipitates from dorsal horn were probed for either β-ENaC, *n* = 3 (IgG lane is cut) or (**f**) γ-ENaC subunits, *n* = 3 (arrows indicate full-length and cleaved proteins). α-Tubulin loading controls are shown. (**g**) Cav3.2 immunoprecipitates from lumbar dorsal root ganglia (L3-L6) were probed for either β-ENaC, *n* = 3 or (**h**) γ-ENaC subunits, *n* = 3 (arrows indicate full-length and cleaved proteins). α-Tubulin loading controls are shown

Because there are several reports in the literature regarding the overlapping expression of both ENaC and Cav3.2 calcium channels in the brain, we first probed Cav3.2 immunoprecipitates from whole brain lysates with either α-, β-, or γ- ENaC antibodies. β- and γ- ENaC subunits bound to Cav3.2 channels were consistently detected (Fig. 1a-d). In contrast, the α-ENaC antibody did not detect full-length or cleaved α-ENaC subunits (Fig. 1a). If ENaC sodium channels interact with Cav3.2 calcium channels in the nervous system, then we might be able to detect channel complexes in peripheral neuronal tissues, where Cav3.2 calcium channels are expressed abundantly and play a role in peripheral pain transmission. Furthermore, β- and γ-ENaC subunits, but not α-ENaC, have been shown to be expressed in dorsal root ganglia (DRG) at both protein and messenger levels [11]. Accordingly, we detected β-ENaC and γ-ENaC subunits bound to Cav3.2 immunoprecipitates from mouse lumbar DRGs (L4-L6) and dorsal horns (Fig. 1e-h).

**Fig. 2:**
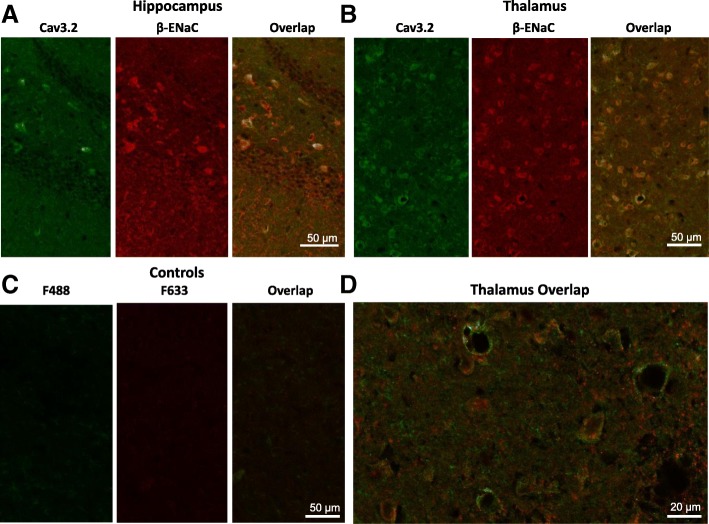
Brain slices showing colocalization of Cav3.2 and β-ENaC in the thalamus and hippocampus. Staining of brain slices with mouse Cav3.2 (green) and polyclonal β-ENaC (red) antibodies. Four mouse brain slices were analyzed in each case. (**a**, **b**) Hippocampal (**a**) and thalamic (**b**) slice confocal images stained with Cav3.2 (green) or β-ENaC (red) antibodies are shown. (**c**) Negative controls using secondary antibodies only (Alexa F488 (green) and Alexa F633 (red)) are shown. (**d**) Larger magnification of immunostaining of thalamic cells stained with both antibodies

Next, we evaluated if Cav3.2 calcium channels expressed in certain regions of the brain may show overlapping expression with ENaC. We stained mouse brain slices with Cav3.2 and β-ENaC antibodies and assessed the overlap in expression of the two proteins (Fig. 2a-d). For example, in the thalamus overlapping expression could be observed in 261 out of 276 cells examined whereas control slices did not yield a fluorescence signal (Fig. 2b, c, d). Similarly, we detected a strong co-localization in the hypothalamus where 591out of 618 cells co-stained for both Cav3.2 calcium channels and β-ENaC subunits (data not shown). Overlapping expression was also detected in the hippocampus (Fig. 2a).

These findings altogether suggest that Cav3.2 and ENaC show overlapping expression in the thalamic region, and that there are protein complexes between Cav3.2 calcium channels and β-ENaC subunits.

### The N-terminus region of β-ENaC contributes to Cav3.2 interactions

**Fig. 3:**
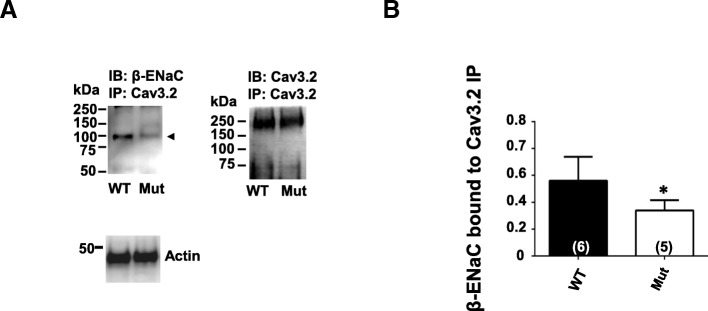
β-ENaC N-terminus lysines contribute to Cav3.2interactions. (**a**) Cav3.2 immunoprecipitates from tsA201 cells transfected with either wild type (WT) β-ENaC or mutant (Mut) β-ENaC (K4R/ K5R/ K9R/ K16R/ K23R) subunits were probed for β-ENaC, *n* = 5–6. Membranes were stripped and probed for Cav3.2 by Western blot. Actin loading controls are shown, *n* = 5–6. (**b**) Quantification analysis of β-ENaC bound to Cav3.2 immunoprecipitates

Next, we asked if the N-Terminus tail of β-ENaC is responsible for the channel complex formation, more specifically if the lysines present in this intracellular region of β-ENaC are involved. We expressed WT or mutant β-ENaC subunits (K4R, K5R, K9R, K16R, K23R) in CAD cells and performed co-immunoprecipitations with this neuronal derived cell line. Binding of the mutant β-ENaC subunit to endogenous Cav3.2 channels was reduced by 50% compared to WT β-ENaC (Fig. 3a, b). This result strongly suggests that the cluster of lysines present in the N-terminus of β-ENaC is partly responsible for the formation of the Cav3.2 / ENaC channel complex, but it is likely that other regions of β-ENaC are also involved.

### Cav3.2 and ENaC subunit affect each other’s cell surface expression

**Fig. 4:**
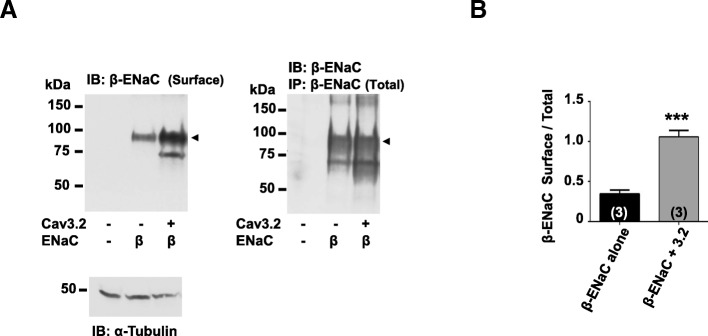
Cav3.2 calcium channels promote trafficking of β-ENaC subunits to the cell surface. (**a**) Western blot showing β-ENaC surface and total pools from tsA201 cells labelled with biotin and transfected with either Cav3.2 alone or Cav3.2 + β-ENaC subunits, *n* = 3. (**b**) Quantification of β-ENaC surface pool levels. An α-tubulin loading control is shown

Next, we asked if β-ENaC subunit trafficking to the cell surface may be affected by co-expression of Cav3.2 channels in tsA cells. We detected a 2.5 fold increase in β-ENaC subunit surface pool (Fig. 4) when Cav3.2 channels were co-expressed. This result suggests that the formation of the Cav3.2/β-ENaC complex either traffics more effectively to the cell surface, or that β-ENaC is stabilized in the plasma membrane once incorporated into a complex with Cav3.2.

**Fig. 5:**
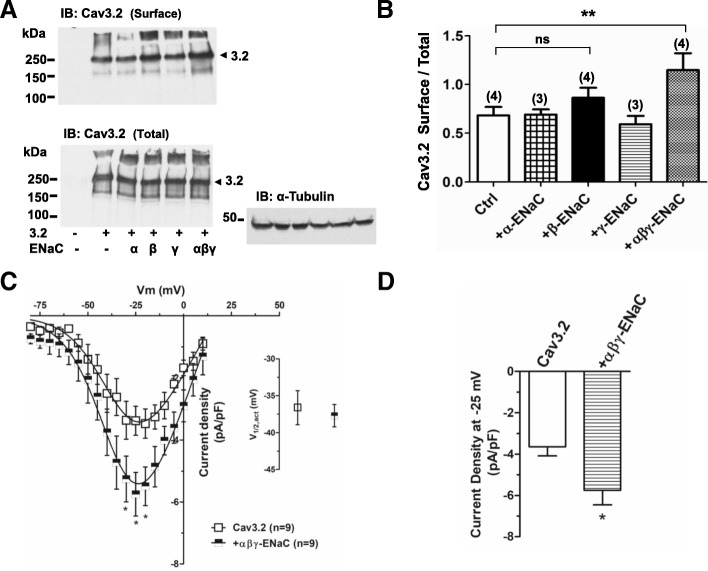
αβγ-ENaC mediated effects on Cav3.2 surface pool and calcium currents. (**a**) αβγ-ENaC increases the Cav3.2 surface pool in tsA201. Cells were labelled with biotin and transfected with either Cav3.2 alone or Cav3.2 + α-, β-, or γ- ENaC subunits individually or Cav3.2 + αβγ-ENaC fully assembled channels, n = 3–4. An α-tubulin loading control is shown. (**b**) Quantification analysis of Cav3.2 surface pool. Statistical analysis was performed with ANOVA. (**c**) αβγ-ENaC significantly increases Cav3.2 peak current density. Average current densities (pA/pF) as a function of voltage in CAD cells transfected with empty plasmid (open squares) or αβγ-ENaC (black and white squares). Inset: voltage for half activation. (**d**) Peak current density measured at − 25 mV in absence or presence of αβγ-ENaC. Cells were incubated with 100 nM amiloride which was washed off before experimentation

We next asked if ENaC promotes a reciprocal trafficking of Cav3.2 to the plasma membrane. We performed surface biotinylation assays coupled with western blot analysis. We did not detect a significant increase in the surface pool of Cav3.2 channels (Fig. 5a, b) when β-ENaC or γ-ENaC subunits were individually co-expressed with calcium channels in tsA-201 cells. In contrast, there was a 2-fold increase in Cav3.2 cell surface expression when all three αβγ-ENaC subunits were co-expressed with the calcium channels (Fig. 5a, b). These results suggest that fully assembled αβγ-ENaC channels can increase the number of Cav3.2 calcium channels at the plasma membrane, at least in tsA-201 cells.

### Functional effects on Cav3.2 channel currents

To investigate the effects of fully assembled αβγ-ENaC channels on Cav3.2 current activity, we first coexpressed Cav3.2 channels and αβγ-ENaC channels in tsA-201 cells. However, we noted very large leak currents that were toxic to the cells, and that could not be fully inhibited by adding the ENaC blocker amiloride to the cell culture medium. Instead, we compared whole cell currents recorded from cells expressing Cav3.2 channels in absence and presence of β-ENaC subunits which by themselves do not support ionic currents. We did not find any effect on the voltage-dependence of activation and whole cell current density (Additional file 1: Figure S1A and inset), or steady state inactivation (Additional file 1: Figure S1B and inset) of Cav3.2 channels. There was also no effect on activation and inactivation kinetics (data not shown). The absence of an effect on whole cell current density is consistent with the cell surface biotinylation experiment shown in Fig. 5b.

To ascertain if fully assembled ENaC channels may affect Cav3.2 channel activity, we instead transfected CAD cells which endogenously express Cav3.2. Cells were grown in medium supplemented with amiloride, which was then washed off prior to recordings. Currents were elicited every 5 mV from a holding potential of − 100 mV to test different voltages. Figure 5c shows average current densities (pA/pF) as function of membrane voltage, revealing that αβγ-ENaC expressing cells show significantly larger T-type currents compared to those of the control condition (*p* < 0.05). These data strongly implicate αβγ-ENaC in regulating macroscopic T-type current amplitude (Fig. 5c, d). There was, however, no effect on the half-activation potential (Fig. 5c inset).

Altogether, these experiments strongly suggest that fully assembled αβγ-ENaC channels are capable of modulating Cav3.2 calcium currents.

## Discussion

The present study reveals the existence of a protein complex between Cav3.2 calcium channels and ENaC in the central and peripheral nervous systems. We detected robust co-expression of this channel complex in neurons from the thalamus, hypothalamus, hippocampus, lumbar dorsal root ganglia and dorsal horn by means of immunohistochemistry and co-immunoprecipitation assays. We also identified a cluster of lysines present in the cytosolic N-terminus tail of β-ENaC (K4R/ K5R/ K9R/ K16R/ K23R, mouse sequence) that is in part responsible for the formation of this complex with Cav3.2 channels. Co-expression of Cav3.2 channels with αβγ-ENaC resulted in a significant increase in peak current density, demonstrating a functional role for the Cav3.2/ αβγ-ENaC interaction. The increased current density, without apparent changes in current kinetics, in conjunction with increased cell surface abundance of Cav3.2 channels when αβγ-ENaC was co-expressed suggests an increase in the numbers of Cav3.2 channels in the plasma membrane. This could be mediated either by an increase in forward trafficking, or by an increase in the stabilization of the channels in the plasma membrane. We do not know how ENaC mediates this effect, however, one possibility may be through an action on the ubiquitination levels of Cav3.2, which we have previously shown to potently regulate cell surface expression of these channels [13].

Although β-ENaC subunits can mediate this interaction, we did not observe any difference in current density when Cav3.2 channels were examined in the presence of β-ENaC subunits, suggesting that fully assembled αβγ-ENaC channels are required for this effect. On the other hand, β-ENaC cell surface expression was enhanced in the presence of Cav3.2 suggesting that the β-ENaC subunit is sufficient for T-type channel interactions. We did not examine analogous effects on γ-ENaC subunit expression in tsA-201 cells, but we do note that this subunit has similar positively charged amino acids present in its cytosolic N-terminus tail such as K6, K8, K10, K12, K13, K26 (mouse sequence). In contrast, the α-ENaC N-terminus only possesses one lysine K23 from which one might predict a lack of interaction with Cav3.2 channels. Although β- and γ-ENaC subunits consistently co-immnoprecipitate with Cav3.2 calcium channels from different neuronal tissues, we do not exclude the possibility that other protein partners are directly involved in the formation of Cav3.2 / ENaC complexes. For example, we have recently shown that Cav3.2 calcium channels can be clustered at the plasma membrane via spectrin / ankyrin B binding [26]. Spectrin is a highly abundant cytoskeletal protein in the nervous system [27, 28] and ENaC can also bind to both ankyrin and spectrin [29, 30], suggesting these cytoskeletal elements as a possible link between these channels. What is, however, curious is that we observed functional effects of αβγ-ENaC on Cav3.2 channels in CAD cells, whereas we could not detect co-immunoprecipitation of α-ENaC with Cav3.2 in brain tissue. This is not due to a lack of expression of α-ENaC itself in the brain (see control lane in Fig. 1a). It is important to note that the functional effects on Cav3.2 channels in CAD cells involved the exogenous and thus artificial expression of all three ENaC subunits, and it is possible that α-ENaC expression in the brain may be restricted to neurons that may not endogenously express high levels of Cav3.2, thus accounting for our inability to co-immunoprecipiate Cav3.2 with α-ENaC. Alternatively, there are several splice isoforms of α-ENaC and it is possible that Cav3.2 channels selectively interact with a variant that is not picked up with the used antibody which is directed against an epitope in the N-terminus where some splice variation can occur. It is also interesting to note that sensory neurons appear to only express β- and γ- ENaC subunits [11], thus raising the question as to what the function of these subunits may be in the absence of detectable levels of α-ENaC, at least under normal physiological conditions. It is possible that α-ENaC expression might be induced under certain pathological conditions.

Interestingly, Younger and colleagues recently described that presynaptic DEG/ENaC channels made of pickpocket11 (PPK11) and pickpocket16 (PPK16) genes are responsible for both the acute induction and long term expression and maintenance of homeostatic synaptic plasticity at the neuromuscular junctions of *Drosophila* motor neurons [31]. In their proposed model these authors suggested that the increased sodium influx leads to a change in presynaptic resting potential which in turn directly or indirectly leads to an increase in presynaptc calcium influx via Cav2.1 calcium channels. This example of ENaC and Cav2.1 channels working in concert to maintain the homeostatic synaptic plasticity raises the possibility that interactions between ENaC and Cav3.2 channels could also be important for fine tuning synaptic activity. This may include the afferent pain pathway where Cav3.2 channels have been shown to regulate synaptic activity in the dorsal horn [12, 13, 32] and hippocampal circuits where Cav3.2 channels have been shown to fine tune NMDA receptor mediated synaptic transmission [33].

It is also important to reiterate that ENaC channel activity in kidney cells is regulated by intracellular calcium ions [22]. It is known that the kidney expresses T-type calcium channels [34] and it is therefore conceivable that Cav3.2 channels could provide the calcium source needed for calcium dependent regulation of ENaC channels. Experiments designed to examine the effects of Cav3.2-mediated calcium entry on ENaC channel function will provide insights into such a possibility.

Altogether, we have identified the existence of a protein complex involving Cav3.2 and ENaC channels. Further work will be needed to elucidate the physiological significance of this interaction in neuronal and perhaps non neuronal tissues.

## Additional file

Additional file 1: **Figure S1.** β-ENaC does not modify Cav3.2 currents or biophysical parameters. (DOCX 195 kb)

## Abbreviations

CAD cell: Cath (catecholaminergic)-a-differentiated
CFTR: Cystic Fibrosis transmembrane conductance regulator
DRG: Dorsal root ganglia
ENaC: Epithelial Sodium Channel
NMDA: N-methyl-D-aspartate
PPK11: Pickpocket11
PPK16: Pickpocket16
