# Supplementary material for: Cav3.2 calcium channel interactions with the epithelial sodium channel ENaC
Source: Mol Brain. 2019 Feb 8;12:12. doi: 10.1186/s13041-019-0433-8 (PMC6368719; doi:10.1186/s13041-019-0433-8)
Supplement: Supplementary file 1 — Figure S1. β-ENaC does not modify Cav3.2 currents or biophysical parameters. (DOCX 195 kb) [file 13041_2019_433_MOESM1_ESM.docx]

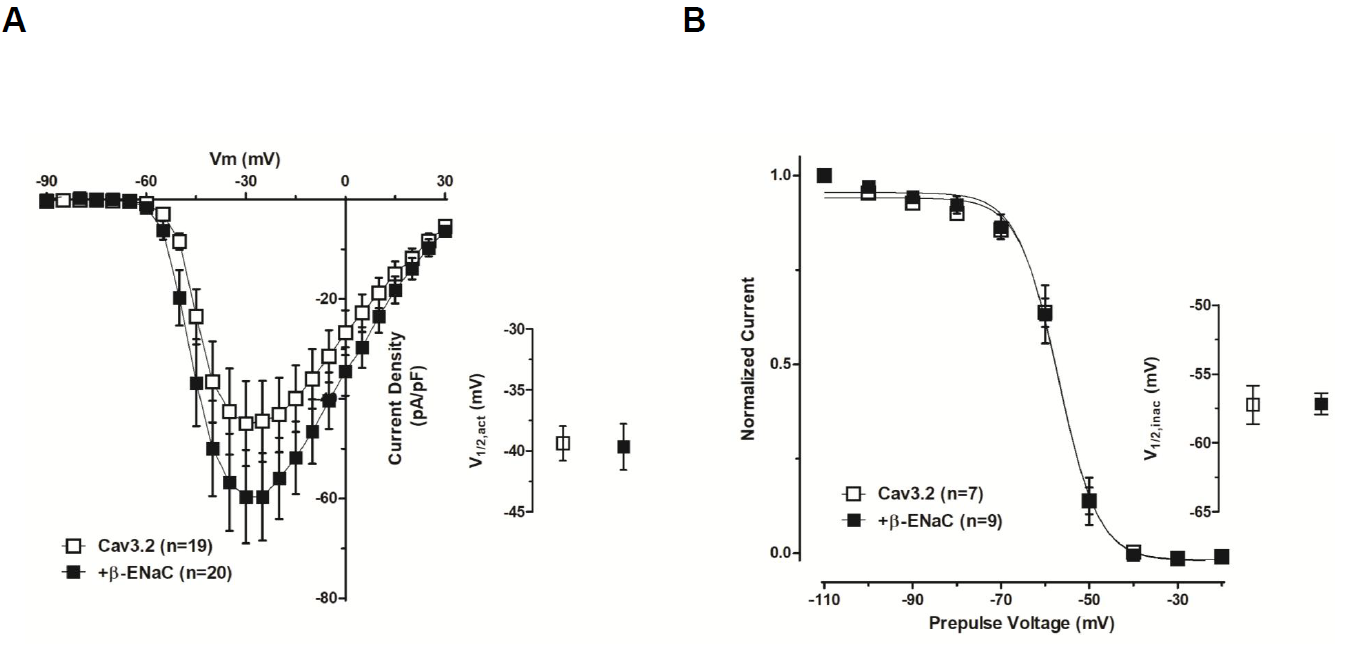


**Figure S1** β-ENaC does not modify Cav3.2 currents or biophysical parameters. (A) Average current densities (pA/pF) as a function of voltage in tsA-201 cells transfected with Cav3.2 channels in the absence (open squares) and presence of β-ENaC (filled squares). Inset voltage for half activation. (B) Steady-state inactivation curves for Cav3.2 channels in the absence (open squares) and presence of β-ENaC (close squares). Inset: voltage for half inactivation.
